# Effects of Formulation and Extrusion Conditions for Isolated Pea Protein-Based High-Moisture Meat Analogs: Insights into Gelation and Structural Development

**DOI:** 10.3390/gels12010042

**Published:** 2026-01-01

**Authors:** Yu Zhang, Hyun-Woo Choi, Yunju Lee, Gi-Hyung Ryu, Bon-Jae Gu

**Affiliations:** 1 Department of Food and Quality Engineering, Nanning University, Nanning 530200, China; 2 Department of Food Science and Technology, Food and Feed Extrusion Research Center, Kongju National University, Yesan 32439, Republic of Korea

**Keywords:** isolated pea protein, gelation, formulation, process variables, meat analog

## Abstract

This study examines how varying the isolated pea protein (IPP) levels (0, 10, 20, 30, 40, 50%) together with key extrusion conditions, including moisture level, barrel heating profile, and screw rotation speed, affect the physicochemical attributes and textural characteristics of high-moisture meat analogs (HMMAs). Results indicated that increased IPP content reduced the fiber structure, springiness, cohesiveness, chewiness, cutting strength, and integrity index of HMMAs. Processing conditions resulted in pronounced changes in both the physicochemical attributes and texture of HMMAs. The increase in moisture content resulted in a decrease in HMMA fiber structure and textural properties. In contrast, increases in barrel temperature and screw speed were associated with higher TPA values, greater cutting strength in both vertical and parallel orientations, and an improved integrity index in HMMAs. Furthermore, the gelation behavior of IPP played a critical role in the formation of the fibrous structure, with optimal gel strength and water retention achieved under specific extrusion conditions. These findings underscore the importance of protein gelation in structuring IPP-based meat analogs and provide insights into the gel-based mechanisms underlying their textural properties. Overall, the optimum IPP content to produce HMMAs in this experiment was 30%, and the process variables were 55% moisture content, barrel temperature of 160 °C, and screw speed of 250 rpm.

## 1. Introduction

Meat analogs are plant-based meats or artificial meats that are composed of plant proteins and other non-animal ingredients to make them mimic the taste, texture, and appearance of traditional meat products [1]. As the world population is increasing, the demand for meat is also increasing. Conventional livestock farming is detrimental to the environment [2]. In addition to reducing the greenhouse gas emissions, considerable water resources are saved, and land-use is reduced with plant-based meat. Meat consumption, particularly uncooked or undercooked meat, is one of the likely causes of acquiring zoonotic diseases [3]. Moreover, a significant amount of red meat consumption is linked with increasing the chances of cardiovascular diseases, cancer, and other diseases [4]. Plant-based meat typically has lower content of saturated fat and cholesterol. This signifies that plant-based meat analog requires an essential need to be developed and the quality must be restructured. Presently, in the market, many plant-based meat analog products are available such as burgers, sausages, chicken, beef, and seafood substitute items that have gained market value and attracted consumers [5].

The food technology has been developed for so much that recently, it makes not only the texture but the taste of the plant-based meat products in the resemblance of the traditional meat. The used technologies are extrusion technology [6], cell culture technology [7], electrospinning technology [8], and 3D printing technology [9]. Of these, the most common approach used to produce meat analogs is extrusion technology. The high-moisture extrusion technology applies a twin-screw extruder under high temperature and pressure conditions to cook and mix plant proteins (soy protein, pea protein) with water, oil, and other additives. It sets up a fibrous structure with mechanical shear force and heat treatment [10]. Meanwhile, the moisture content of the raw materials is over 40% under the high-moisture extrusion, and the proteins denature and reorganize under high temperature and pressure to form a dense fibrous network structure [11]. The extruded material is then subject to rapid cooling through a cooling die to fix the fibrous structure, leaving a meat-like texture in the product [12].

The formation of a gel-like network during high-moisture extrusion is essential for mimicking the fibrous texture of meat. Protein gelation, driven by thermal denaturation and shear-induced alignment, is a key mechanism in the development of meat analogs with desirable chewiness and water-holding capacity. Understanding the gelation properties of plant proteins, such as pea protein, is therefore crucial for optimizing the texture and stability of high-moisture meat analogs.

Plant protein ingredients used in making meat analogs mainly include soy protein, pea protein, wheat gluten, rice protein, and faba bean protein, with soy protein being the most widely used. Soy protein features a high-quality protein and an amino acid profile close to that of animal protein [13]. However, soy is a frequent allergen and can give sensory discomfort to some consumers [14]. Thus, pea protein is increasingly used in the production of meat analogs with a high-protein content, possessing excellent functional and nutritional properties [15]. Peas are an easily grown, versatile crop. Pea protein is characterized by high-protein content and a good amino acid profile, in particular, a high lysine content [16], in comparison to a major part of other plant proteins. Pea protein is also less allergenic than soy and does not contain common allergens [17]. Thus, this product is more suitable for a large percentage of the population. Additionally, pea protein has a neutral flavor, so it works well during production, while good compatibility in the blending of other formulation components occurs without influencing the final taste of the product, which is of high importance for food processing.

Currently, several works have reported pea protein as having great potential and advantage in making meat analogs. Osen et al. [18] analyzed three commercially available pea protein isolates and reported that, despite having comparable overall chemical compositions, differences in their functional behavior altered the viscosity of protein aggregates during the early heating stage of extrusion. The results of Schreuders et al. [19] showed that pea protein-gluten mixtures show potential for the preparation of structured plant protein materials, but their areas of application may be different from the potential areas of application of soy protein-gluten mixtures. However, the systematic optimization of raw material ratios and extrusion process variables to obtain the desired response of high-moisture meat analogs based on isolated pea protein (IPP) had not been reported. Therefore, this study systematically investigates the gelation-structure relationship in isolated pea protein (IPP) during HMEC, with a focus on elucidating how protein concentration and process parameters (moisture, temperature, shear) jointly govern the transition from a gel-like network to an anisotropic fibrous structure. By comparing the behavior of IPP with the well-established soy protein (ISP) under identical processing windows, we aim to provide mechanistic insights into the specific gelation limitations of pea protein and identify process-driven strategies to overcome its textural shortcomings, which have not been comprehensively addressed in previous comparative or optimization studies. Thus, the experiment was designed to determine the most suitable IPP inclusion level (0–50%) for high-moisture meat analogs and to optimize key extrusion parameters such as moisture content, barrel temperature, and screw speed. Therefore, based on the results, the optimum production formula and process conditions will be determined, laying the foundation for subsequent development and production of high-moisture meat analog products of IPP.

## 2. Results and Discussion

### 2.1. Fiber Structure

**Figure 1:**
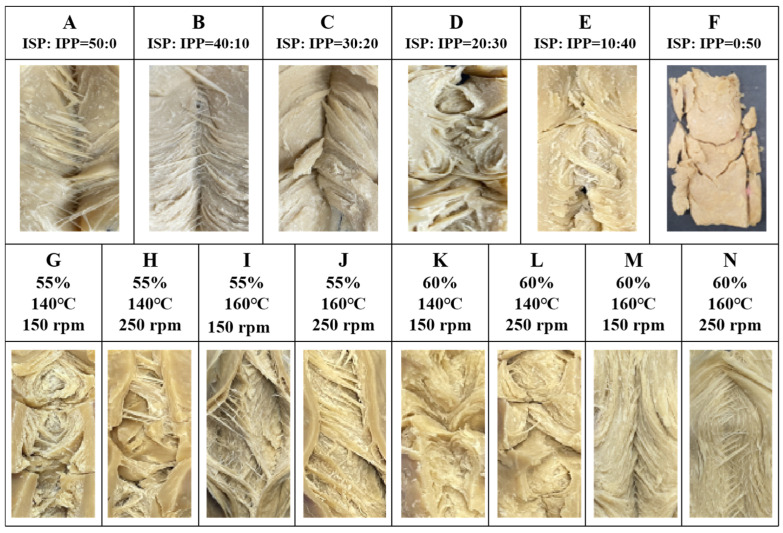
Fiber structure of HMMA according to isolated pea protein content (**A**–**F**) and process variables (**G**–**N**).

The fiber structure of high-moisture meat analogs (HMMAs) is influenced by the content of isolated pea protein (IPP), as shown in Figure 1A–F. During the high-moisture extrusion cooking (HMEC) process, the long cooling die suppressed the expansion of HMMAs, and no bubbles like those in low-moisture meat analogs (LMMAs) were observed [20], forming a dense and clearly layered fiber structure. As shown in Figure 1A–F, the higher the ISP content, the more obvious the fiber structure, while the higher the IPP content, the less the fiber structure and degree of organization of HMMAs. In this experiment, the highest IPP content that can achieve tissue organization was 40%. HMMA with 50% IPP added did not form a tissue structure, so it was excluded from the analysis experiment (Figure 1F).

This observed concentration-dependent decline in fibrous structure with increasing IPP content suggests a distinct gelation and network formation behavior compared to soy protein. We hypothesize that this phenomenon arises from two interconnected mechanisms. First, pea protein exhibits a higher critical concentration requirement for forming a continuous, self-supporting gel network under high-moisture extrusion conditions. Beyond approximately 40% IPP, the gel strength becomes insufficient to withstand the mechanical shear and flow alignment within the extruder, leading to structural collapse, as evidenced by the 50% IPP sample. Second, the interplay between thermal denaturation and shear-induced alignment, which effectively promotes fibrous structure in ISP, appears less efficient for IPP at equivalent concentrations. The failure of the 50% IPP sample to form a coherent structure points to a synergistic limitation involving both formulation and process. From a formulation perspective, exceeding approximately 40% IPP likely surpasses the critical concentration for effective network percolation under the given conditions due to IPP’s inherent weaker gelation capacity compared to ISP. From a process perspective, the standard thermal-mechanical energy input (150 °C, 250 rpm, 55% moisture) applied uniformly across all formulations may be insufficient to adequately denature, align, and cross-link the high concentration of pea protein to form a self-supporting network. Therefore, it is not solely a formulation limit but rather indicates that successful texturization of high IPP concentrations would require specifically tailored process adjustments (e.g., higher temperature/shear) to compensate for its gelation deficiency. In this context, the distinction between a ‘gel-like’ and a ‘fibrous’ structure is primarily based on macroscopic and textural observations. A ‘gel-like’ structure refers to a more homogeneous, isotropic matrix with high water-holding capacity but lower mechanical anisotropy and visible layering, often associated with higher moisture content and/or insufficient shear alignment. In contrast, a ‘fibrous’ structure is characterized by visible, oriented layers or strands in the product’s cross-section (Figure 1), coupled with higher cutting strength anisotropy (difference between vertical and parallel direction) and superior chewiness.

The effect of process variables on the fiber structure of HMMA with 30% IPP added is shown in Figure 1G–N. During the HMEC process, the fiber structure of IPP-based HMMA decreased with increasing moisture content, while it increased with increasing barrel temperature and screw speed. According to Choi and Ryu [21], the fiber structure of ISP increased with increasing moisture content during HMEC, resulting in improved texturization. The increase in moisture content during HMEC leads to a more uniform distribution of moisture in meat analogs, which interacts with protein molecules and promotes protein solubilization and incorporation, making the proteins more likely to form a gel-like structure rather than a fibrous structure. The barrel temperature and screw speed can increase the movement and collision frequency of protein particles, promoting protein interaction and cross-linking. This cross-linking contributes to the formation of a tighter network structure, resulting in an increased fiber structure in the meat analogs [22]. In addition, the increase in barrel temperature helps to accelerate the protein solubilization process, making it easier for proteins to form a fibrous structure during extrusion. Meanwhile, increasing the screw speed can increase the mechanical shear force inside the extruder, which helps to disperse the protein uniformly in water and oil, and further promotes the dissolution of protein and the formation of a fibrous structure. The observed reduction in fiber formation with increasing IPP content can be attributed to the inferior gelation capacity of pea protein compared to soy protein. IPP requires higher concentrations and specific thermal conditions to form stable gels, which influences its ability to develop a continuous protein network during extrusion [23]. This gelation behavior directly impacts the structural integrity and textural quality of the final product. In addition, the constant presence of wheat gluten and corn starch provided a consistent structural background and moisture environment against which the effects of IPP substitution and process variables could be isolated and evaluated. The viscoelastic network contributed by WG likely underpinned the baseline integrity of all samples, while CS modulated the local aqueous phase, collectively influencing the final gelation and fiber formation dynamics of the blended proteins.

### 2.2. Texture Profile Analysis (TPA) and Cutting Strength

Springiness describes how much the HMMA sample recovers its original form once the applied force is released. Cohesiveness reflects the internal resistance of the sample to deformation, and chewiness represents the amount of energy needed to chew the product [24]. In contrast, cutting strength refers to the force required to sever the HMMA. The impact of IPP content on the TPA and cutting strength of HMMA is shown in Table 1. Both elasticity, cohesiveness, and chewiness decrease with increasing IPP content. As mentioned earlier, IPP gelation is relatively weaker compared to ISP, allowing ISP to form a gel-like structure more effectively under high-temperature and high-pressure extrusion conditions, thereby increasing the elasticity and cohesiveness of HMMA. Additionally, an increase in IPP content in HMMA also leads to a decrease in cutting strength. This trend agrees with Schreuders et al. [19], who reported that pea protein-based systems exhibit lower mechanical strength and a narrower processing window for fibrous structure formation than soy protein-based blends. Such differences in structuring behavior help explain the reduced elasticity, cohesiveness, and cutting strength observed with increasing IPP content in this study.

Table 2 summarizes how processing parameters influenced the TPA profile and cutting strength of HMMA containing 30% IPP. In these samples, elasticity did not differ noticeably with changes in moisture level or screw speed, whereas increasing the barrel temperature led to a significant rise in elasticity. With an increase in moisture content, both cohesiveness and chewiness of HMMA decrease, while an increase in barrel temperature and screw speed leads to an increase in both. The increase in barrel temperature intensifies the degree of protein denaturation and reformation of new molecular structures, which contributes to the formation of a more stable protein network structure, thereby enhancing the textural properties of HMMA [23,25]. Additionally, higher temperatures help enhance the protein’s gelling ability. ISP and IPP can form stronger gel-like structures at higher temperatures, which maintain better textural properties upon cooling [26]. Cutting strength increases longitudinally and transversely as moisture content decreases and barrel temperature and screw speed increase. At 55% moisture content, HMMA exhibits relatively high chewiness and denser fiber structure, with an improvement in texture profile. However, when the moisture content reaches 60%, cohesiveness decreases, exhibiting a dough-like texture. Similarly, under the same moisture conditions, an increase in barrel temperature from 140 °C to 160 °C results in an increase in both cohesiveness and chewiness, consistent with the findings of Mazaheri et al. [27]. The textural properties of HMMA are closely related to the gel strength formed during extrusion. The decrease in springiness and cohesiveness with higher IPP content indicates weaker gel formation, which is consistent with the lower gelation ability of pea protein. In contrast, the enhancement of these properties with increased barrel temperature and screw speed suggests improved protein gelation through enhanced protein-protein interactions and cross-linking.

### 2.3. Water-Holding Capacity (WHC)

The WHC of HMMA containing 30% IPP is influenced by process variables, as shown in Table 2. The WHC of HMMA is positively correlated with moisture content and negatively correlated with barrel temperature, while the effect of screw speed is not significant. Because of the cooling mold, HMMA suppresses expansion and does not exhibit a structure with air layers, which is its main characteristic and differs from low-moisture meat substitutes [28]. During the HMEC process, an appropriate increase in moisture content can enhance the hydration of protein molecules, forming a more stable colloidal structure, thereby improving WHC [29]. Moreover, with the increase in barrel temperature, the quantitative increase in protein bonds reduces the capillary penetration and diffusion of water molecules, resulting in a decrease in WHC. WHC is a direct indicator of the gel network’s ability to immobilize water. The negative correlation between WHC and barrel temperature may be due to excessive protein denaturation, which compromises the gel matrix and reduces its water-binding capacity. These findings highlight the delicate balance required in process conditions to achieve optimal gel formation and hydration properties.

The relationship between WHC and textural properties in HMMA is complex. As noted in Table 2, WHC was positively correlated with moisture content but negatively correlated with barrel temperature, while key textural parameters like chewiness and cutting strength often showed opposite trends with temperature. This suggests that high WHC does not automatically indicate a strong, elastic gel network. The increase in WHC with higher moisture content may primarily reflect greater physical entrapment of free water within the looser matrix, which can coincide with reduced texture integrity. Conversely, the decrease in WHC at higher barrel temperatures likely results from increased protein aggregation and network densification, which reduces pore size available for water retention but simultaneously enhances mechanical strength through stronger protein-protein interactions. Therefore, in the context of HMEC, moderate WHC coupled with high textural values may be a more reliable indicator of a well-formed, strong gel network than WHC alone.

### 2.4. Integrity Index

The integrity index is a parameter that represents the organization of HMMA, indicating the degree of residual structure of meat analogs after hydration, compression, dispersion, and drying [28]. The effect of IPP content on the integrity index of HMMA is shown in Table 1. As the IPP content increases, the reduction in texturization leads to a decrease in the integrity index, which may stem from differences in gelation ability between the two proteins. According to Zhang et al. [23], unlike soy protein, which can gel at lower concentrations (12%), pea protein requires higher concentrations (14%) to form a gel. This difference in gelation ability may affect the formation of heterogeneous gels between the two proteins during extrusion molding at the same concentration.

The impact of process variables on the integrity index of HMMA containing 30% IPP is shown in Table 2. With an increase in moisture content, the integrity index decreases, while an increase in barrel temperature and screw speed leads to an increase in the integrity index. This is consistent with the results of fiber structure, indicating that the higher the fiber structure of HMMA, the higher its integrity index. Higher moisture content can destabilize the fiber structure of meat analogs, reducing their integrity index. But this is contrary to the results reported by Zhang and Ryu [20] and may be due to the difference in extrusion temperature. An increase in barrel temperature can accelerate the dissolution and cross-linking of proteins, thereby promoting the formation of fiber structures in meat analogs [25,28]. However, excessively high barrel temperatures may lead to excessive protein denaturation and heat inactivation, thereby reducing the integrity index of meat analogs [23]. Additionally, an appropriate increase in screw speed contributes to the formation of a more uniform and stable structure, enhancing the integrity index. The integrity index reflects the stability of the gel network under harsh conditions. The decrease in integrity with higher moisture content suggests a dilution effect on protein concentration, impairing gel formation. Conversely, higher temperatures and screw speeds promote stronger gel networks, enhancing the structural resilience of the meat analog.

### 2.5. Nitrogen Solubility Index (NSI)

**Figure 2:**
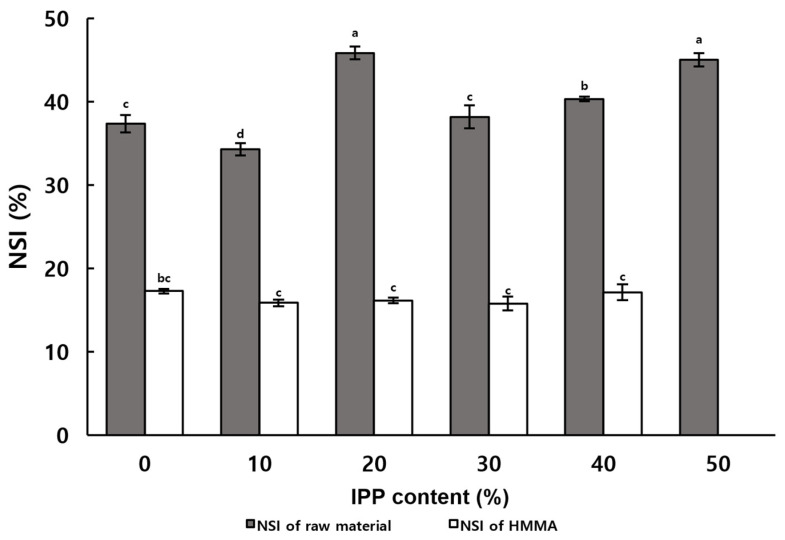
Nitrogen solubility index of the extruded meat analogs prepared with varying IPP contents. Values with different letters in the same column indicate significant differences (*p* < 0.05) by Duncan’s multiple range test.

The NSI is a measure of the degree of protein denaturation [30], which can indirectly indicate the degree of organization of HMMA. Figure 2 demonstrates the difference between the NSI of raw materials and HMMA with different IPP contents. The NSI of the mixed raw materials showed an increasing trend with the increase in IPP content, which indicated that IPP contained more water-soluble nitrogenous proteins than ISP [31]. In contrast, no significant difference was observed in the effect of IPP content on NSI in HMMA. HMMA without added IPP showed a greater difference in NSI before and after extrusion molding compared to HMMA with 50% IPP added, the latter showing more protein denaturation.

The effect of process variables on the NSI of HMMA with 30% IPP added is shown in Table 2. The NSI of HMMA was positively correlated with increasing moisture content, barrel temperature, and screw speed. Wang et al. [25] reported similar results, but O’Kane et al. [26] results showed no significant correlation between the NSI value of HMMA and the barrel temperature. During high-moisture extrusion, an increase in moisture content leads to an increase in moisture in the meat analog, which accelerates protein solubilization and release, and therefore an increase in the nitrogen solubility index [32]. Higher barrel temperatures and screw speeds contribute to the movement and interaction between protein molecules as well as increasing the mechanical shear and compression forces inside the extruder, respectively, making proteins more soluble in water [33]. Changes in the NSI due to changes in the process variables of IPP were quantified by quantifying the water-soluble proteins denatured during extrusion molding, and these data can be used to indirectly estimate the organization of HMMAs. These data can be used as basic data for chemical changes in the structural changes in the replacement meat using IPP, but further studies beyond the water-soluble nitrogen index are needed to gain insight into the mechanisms underlying the structural properties of the replacement meat. NSI provides insight into the extent of protein denaturation and solubility, which are indicative of gelation potential. The positive correlation between NSI and process variables suggests that controlled protein solubilization is essential for initiating gel formation. However, excessive denaturation may lead to over-aggregation, negatively affecting gel quality.

## 3. Conclusions

This study demonstrated that the addition of isolated pea protein (IPP) and the manipulation of extrusion process variables significantly affect the quality of high-moisture meat analogs (HMMA). Higher IPP content generally reduced the structural integrity and texturization of the meat analogs, while process variables such as barrel temperature and screw speed played a critical role in enhancing the textural properties. The increase in moisture content resulted in a decrease in HMMA fiber structure and a decrease in textural properties. The barrel temperature and screw speed, on the other hand, were positively associated with textural measurements, including TPA values and bidirectional cutting strength, as well as with the integrity index of the HMMAs. This study highlights the critical role of protein gelation in determining the structural and textural properties of pea protein-based high-moisture meat analogs. The gel-forming ability of pea protein, though inferior to soy protein, can be optimized through careful control of extrusion parameters to achieve desirable meat-like characteristics. It should be noted that the interpretations regarding protein gelation behavior in this study are primarily inferred from macroscopic textural and structural properties (e.g., TPA, integrity index, fiber formation). While these indicators are well-established correlates of gel network strength and quality in extruded meat analogs, direct evidence from rheological measurements (e.g., small-amplitude oscillatory shear) or thermal analysis (e.g., differential scanning calorimetry, DSC) would provide more fundamental insights into the gelation temperature, gel point, and viscoelastic properties of the IPP systems under study. The inclusion of such analyses in future work will be crucial to unequivocally validate the proposed mechanisms of gelation limitation and process-induced enhancement for pea protein. Nevertheless, the consistent trends observed across multiple physical metrics in the present work offer robust indirect support for the central role of gelation in determining the textural outcome of IPP-based HMMA. Furthermore, while this study focused on physicochemical and structural properties, the nutritional profile, specifically in vitro protein digestibility and the fate of anti-nutritional factors during extrusion, remains an important aspect for the comprehensive evaluation of plant-based meat analogs. Future work should incorporate these analyses to elucidate how extrusion processing affects protein bioavailability and overall nutritional quality.

## 4. Materials and Methods

### 4.1. Materials

Four raw materials were used in this experiment: isolated pea protein (IPP), isolated soy protein (ISP), wheat gluten (WG), and corn starch (CS). The ingredients were sourced from Shuangta Food Co., Ltd. (Yantai, China), Tianjing Plant Protein Co., Ltd. (Pingdingshan, China), Roquette Frères (Lestrem, France), and Samyang Corporation (Ulsan, Republic of Korea), respectively. The raw material proportions in the optimization process for the isolated pea protein-based high-moisture meat analogs (Experiment 1) and process variables (Experiment 2) are shown in Table 3. The selection of the IPP content range (0–50%) was designed to systematically evaluate the structural transition from a soy protein-dominated to a pea protein-dominated system while maintaining a constant total protein level (50%) from plant sources. This range covers the full substitution spectrum and allows for the identification of a critical substitution threshold for acceptable texture. The levels of wheat gluten (WG, 40%) and corn starch (CS, 10%) were fixed based on preliminary trials and common practice in HMMA formulations. WG was included as a key structural agent for its viscoelastic and network-forming capacity, which is essential for creating a continuous protein matrix during extrusion. CS served as a filler and water distribution modulator, contributing to texture moderation and process stability. The process parameters, moisture content (55–60%), barrel temperature (140–160 °C), and screw speed (150–250 rpm), were chosen based on standard operating windows for high-moisture extrusion of plant proteins and preliminary experiments indicating that these ranges are critical for inducing protein denaturation, gelation, and fibrous alignment while avoiding excessive degradation or under-processing.

### 4.2. High-Moisture Extrusion Process

**Figure 3:**
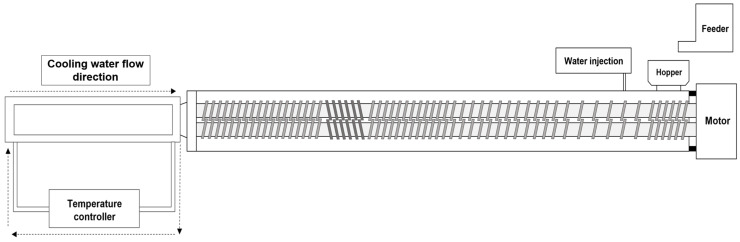
Schematic diagram of high-moisture extrusion system with details of extruder.

A co-rotating twin-screw extruder (model THK31-No. 4; Incheon Machinery Co., Incheon, Republic of Korea) was used to manufacture the high-moisture meat analogs (HMMAs). The screw set in this system has a diameter of 3 cm and an overall length of 69 cm. During the preparation of the meat analogs in the high-moisture extrusion process, a long cooling die was used, and the temperature was maintained at 20 °C by circulating water. The temperature of each barrel zone was adjusted using electric heaters and cooling water. The schematic of twin-screw extruder and screw configuration are presented in Figure 3 and the details of the extrusion process variables are presented in Table 3.

Some of the HMMAs were cut into 1 cm cubic pieces and kept in the fridge for water-holding capacity (WHC), integrity index, texture profile analysis, cutting strength, and degree of texturization analysis. The remaining samples were first frozen at −60 °C and subsequently freeze-dried using a freeze-dryer (Model TFD5505, Ilshin Lab Co., Ltd., Gyeonggi, Republic of Korea) at a chamber pressure of 0.67 Pa, with freeze-drying initiated at −40 °C, followed by grinding to 50–70 mesh powder for nitrogen solubility index (NSI) analysis. The final moisture content of the extruded high-moisture meat analogs was 56.27 ± 2.39%.

### 4.3. Texture Profile Analysis (TPA), Cutting Strength

**Figure 4:**
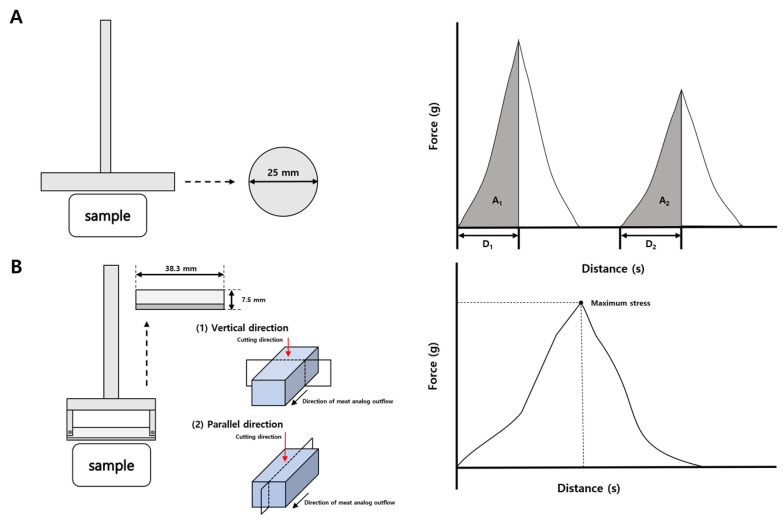
Probe type and force-time curves for springiness, cohesiveness, and chewiness (**A**) and probe type and force-time curves for cutting strength (**B**).

The TPA and cutting strength of the HMMAs with added IPP were measured using the Rheometer (Compac-100II). The measurement probe for the manufactured Sun Sci. Co., Tokyo, Japan, and force-time graph as shown in Figure 4. Springiness, cohesiveness, and chewiness of the HMMA were measured by a 2.5 cm diameter probe up to a maximum stress of 10 kg. The TPA test was conducted at a speed of 80 mm/min, with a 3 s time gap between the two compression cycles. Cutting strength was determined using a 7.5 mm × 38.3 mm blade-type probe, with the load limit set at 2 kg. The deformation speed during the cutting strength test was set to 80 mm/min. Springiness, cohesiveness, and chewiness were calculated using Equations (1)*–*(3) following the method of Trinh and Glasgow [24], and cutting strength was obtained from Equation (4). Ten replicates were collected, after which the two highest and two lowest values were discarded and the mean of the remaining measurements was reported.

Springiness (%) = D_2_/D_1_ × 100 (1)

Here, D_1_ represents the compression distance during the first cycle, while D_2_ denotes the recovered height measured during the second compression.

Cohesiveness (%) = A_2_/A_1_ × 100 (2)

In this equation, A_1_ corresponds to the work performed in the initial compression and A_2_ indicates the work measured during the second compression cycle.

Chewiness (N) = Springiness × Cohesiveness × H(3)

H refers to the peak force recorded during the first compression cycle.

Cutting strength (g/cm^2^) = F_max_/A (4)

F_max_ is the maximum force measured during the cutting test and A is the cross-sectional area of the specimen being cut.

### 4.4. Water-Holding Capacity (WHC)

The method reported by Zhang and Ryu [20] was used to determine the water-holding capacity (WHC) of the HMMAs prepared with IPP. The samples were hydrated in a 50 °C water bath for 16 h, then transferred to a 20-mesh sieve and allowed to drain for 15 min prior to weighing. WHC was then calculated using Equation (5).

WHC (g/g) = (W_b_ − W_a_)/W_a_ (5)

In this equation, W_a_ corresponds to the weight of HMMA in its dried state and W_b_ refers to the weight of the sample following rehydration.

### 4.5. Integrity Index

The integrity index of the IPP-based HMMA was determined using a modified method based on the methods of Samard et al. [28]. First, 4 g of HMMA on a dry matter basis were pressurized in an autoclave at 121 °C for 15 min. After treatment, samples were cooled under running water for 1 min and homogenized for 1 min at 17,450 rpm using a high-speed homogenizer. The homogenized HMMA was washed with running water over a 20-mesh sieve to collect the residues. Residues were collected, dried at 105 °C for 24 h. The integrity index was obtained by expressing the weight of the remaining residues as a percentage of the original sample weight, as calculated using Equation (6):

Integrity index (%) = W_b_/W_a_ × 100% (6)

where W_a_ is the weight of dried HMMA before test and W_b_ is the weight of dried HMMA after test.

### 4.6. Nitrogen Solubility Index

The modified method of Samard et al. [28] was used to estimate the degree of protein denaturation in the IPP-based HMMAs. First, 0.1 g of the ground sample was added to 5 mL of 0.5% KOH solution and stirred for 20 min at 30 °C and 120 rpm to prepare the soluble nitrogen extract. For preparation of the total nitrogen extract, 0.1 g of sample was combined with 2.5 mL of 6 N HCl and hydrolyzed at 100 °C for 24 h. After hydrolysis, the mixture was diluted with 5 mL of distilled water. The extracts were centrifuged at 3000 rpm for 30 min to obtain the supernatant. The determination of the soluble and total nitrogen content is carried out based on the ninhydrin reaction of Starcher [34], and the NSI is calculated by the following Equation (7):

NSI (%) = C_b_/C_a_ × 100% (7)

where C_a_ is the total nitrogen content of HMMA and C_b_ is the soluble nitrogen content of HMMA. The NSI experiment uses the γ-globulin as a standard.

### 4.7. Statistics Analysis

Statistical analyses were conducted using SPSS (version 26.0, IBM-SPSS, Thornwood, NY, USA). A one-way analysis of variance (ANOVA) was applied to evaluate differences among treatments. For items showing significant differences, Duncan’s multiple range test was applied to determine significant differences at the *p* < 0.05 level.

## Figures and Tables

**Table 1:** Effects of IPP content on the texture profile, cutting strength, and integrity index of extruded meat analogs.

Raw Material Content		Springiness (%)	Cohesiveness (%)	Chewiness (g)	Cutting Strength (g/cm^2^)		Integrity Index (%)
ISP ^(1)^ (%)	IPP (%)				Vertical Direction	Parallel Direction	
50	0	92.45 ± 1.78 ^(2) a^	82.26 ± 0.73 ^bc^	5896.77 ± 246.10 ^a^	1205.63 ± 222.33 ^a^	860.36 ± 153.59 ^ab^	84.27 ± 0.49 ^a^
40	10	93.09 ± 0.91 ^a^	81.21 ± 0.35 ^bc^	4696.22 ± 163.35 ^b^	1027.93 ± 28.08 ^b^	933.08 ± 245.35 ^a^	81.26 ± 0.49 ^ab^
30	20	93.35 ± 1.22 ^a^	79.46 ± 2.07 ^cd^	3750.90 ± 337.33 ^c^	818.71 ± 43.86 ^c^	757.22 ± 133.58 ^b^	77.73 ± 1.27 ^abc^
20	30	89.95 ± 1.01 ^a^	74.86 ± 1.97 ^de^	3520.50 ± 193.36 ^c^	608.28 ± 30.27 ^ef^	564.54 ± 80.46 ^c^	76.91 ± 1.89 ^bc^
10	40	83.54 ± 2.96 ^b^	68.00 ± 5.26 ^fg^	2164.87 ± 373.33 ^d^	540.43 ± 30.10 ^f^	359.43 ± 67.86 ^de^	71.65 ± 1.86 ^cd^
0	50	-					

(1) ISP: isolated soy protein, IPP: isolated pea protein. (2) Different letters within a column denote statistically significant differences (*p* < 0.05), as determined by Duncan’s multiple range test.

**Table 2:** Effects of processing conditions on textural attributes, cutting strength, water-holding capacity, and on indices related to integrity and nitrogen solubility in extruded meat analogs.

M.C ^(1)^ (%)	B.T (°C)	S.S (rpm)	Springiness (%)	Cohesiveness (%)	Chewiness (g)	Cutting Strength (g/cm^2^)		WHC (g/g)	Integrity Index (%)	NSI (%)
						Vertical Direction	Parallel Direction			
55	140	150	86.63 ± 0.74 ^(2) c^	70.37 ± 2.14 ^ef^	3804.47 ± 300.51 ^d^	810.12 ± 40.99 ^cd^	669.19 ± 96.05 ^ab^	2.78 ± 0.10 ^d^	77.33 ± 1.17 ^c^	11.76 ± 0.24 ^g^
		250	88.81 ± 2.00 ^bc^	76.16 ± 2.42 ^bcd^	4521.01 ± 227.24 ^c^	910.48 ± 89.45 ^c^	743.05 ± 221.93 ^ab^	2.83 ± 0.04 ^d^	87.50 ± 0.12 ^a^	12.29 ± 0.14 ^efg^
	160	150	92.71 ± 0.43 ^ab^	78.38 ± 0.54 ^abc^	5065.46 ± 142.85 ^b^	1391.11 ± 208.03 ^a^	713.62 ± 258.42 ^ab^	2.65 ± 0.26 ^def^	79.20 ± 0.46 ^bc^	14.80 ± 0.68 ^b^
		250	93.45 ± 0.59 ^a^	82.46 ± 1.25 ^a^	6374.08 ± 240.16 ^a^	1348.74 ± 126.25 ^a^	777.82 ± 86.36 ^a^	2.43 ± 0.12 ^fg^	88.27 ± 0.90 ^a^	13.87 ± 0.57 ^c^
60	140	150	81.63 ± 2.07 ^d^	64.02 ± 1.83 ^g^	1390.91 ± 179.24 ^h^	637.59 ± 58.88 ^ef^	581.02 ± 96.18 ^bcd^	2.85 ± 0.05 ^d^	58.30 ± 0.91 ^h^	14.59 ± 0.08 ^bc^
		250	90.73 ± 1.04 ^ab^	72.19 ± 3.36 ^def^	2839.43 ± 315.75 ^g^	564.46 ± 12.04 ^f^	478.10 ± 116.59 ^cde^	2.81 ± 0.01 ^d^	71.49 ± 0.60 ^d^	16.52 ± 0.11 ^a^
	160	150	91.44 ± 1.23 ^ab^	73.96 ± 1.61 ^cdef^	3180.68 ± 179.38 ^f^	812.28 ± 103.18 ^cd^	609.59 ± 139.45 ^bc^	2.47 ± 0.08 ^efg^	81.03 ± 1.10 ^b^	16.64 ± 0.38 ^a^
		250	93.87 ± 1.25 ^a^	80.19 ± 0.98 ^ab^	3515.33 ± 133.07 ^e^	1109.56 ± 123.43 ^b^	697.74 ± 82.04 ^ab^	2.70 ± 0.12 ^de^	80.70 ± 0.73 ^b^	16.14 ± 0.64 ^a^

(1) M.C: moisture content, B.T: barrel temperature, S.S: screw speed, WHC: water-holding capacity, NSI: nitrogen solubility index. (2) Values with different letters in the same column indicate significant differences (*p* < 0.05) by Duncan’s multiple range test.

**Table 3:** Formulation and operational parameter settings for extrusion cooking designs I and II.

Extrusion Design	Formulation				Operation Parameters			
	IPP ^(1)^ (%)	ISP (%)	WG (%)	CS (%)	MC (%)	BT (°C)	SS (rpm)	Feed Rate (g/min)
Experiment 1	0	50	40	10	55	150	250	100
	10	40						
	20	30						
	30	20						
	40	10						
	50	0						
Experiment 2	30	20	40	10	55	140	150	
							250	
						160	150	
							250	
					60	140	150	
							250	
						160	150	
							250	

(1) IPP: isolated pea protein, ISP: isolated soy protein, MC: moisture content, BT: barrel temperature, SS: screw speed.

## Data Availability

The original contributions presented in this study are included in the article. Further inquiries can be directed to the corresponding author.
